# PHB Biosynthesis Counteracts Redox Stress in *Herbaspirillum seropedicae*

**DOI:** 10.3389/fmicb.2018.00472

**Published:** 2018-03-15

**Authors:** Marcelo B. Batista, Cícero S. Teixeira, Michelle Z. T. Sfeir, Luis P. S. Alves, Glaucio Valdameri, Fabio de Oliveira Pedrosa, Guilherme L. Sassaki, Maria B. R. Steffens, Emanuel M. de Souza, Ray Dixon, Marcelo Müller-Santos

**Affiliations:** ^1^Department of Biochemistry and Molecular Biology, Universidade Federal do Paraná, Curitiba, Brazil; ^2^Department of Molecular Microbiology, John Innes Centre, Norwich, United Kingdom; ^3^Department of Clinical Analysis, Universidade Federal do Paraná, Curitiba, Brazil

**Keywords:** polyhydroxyalkanoates, Fnr, redox regulation, bacterial signal transduction, transcriptomics

## Abstract

The ability of bacteria to produce polyhydroxyalkanoates such as poly(3-hydroxybutyrate) (PHB) enables provision of a carbon storage molecule that can be mobilized under demanding physiological conditions. However, the precise function of PHB in cellular metabolism has not been clearly defined. In order to determine the impact of PHB production on global physiology, we have characterized the properties of a Δ*phaC1* mutant strain of the diazotrophic bacterium *Herbaspirillum seropedicae*. The absence of PHB in the mutant strain not only perturbs redox balance and increases oxidative stress, but also influences the activity of the redox-sensing Fnr transcription regulators, resulting in significant changes in expression of the cytochrome *c*-branch of the electron transport chain. The synthesis of PHB is itself dependent on the Fnr1 and Fnr3 proteins resulting in a cyclic dependency that couples synthesis of PHB with redox regulation. Transcriptional profiling of the Δ*phaC1* mutant reveals that the loss of PHB synthesis affects the expression of many genes, including approximately 30% of the Fnr regulon.

## Introduction

*Herbaspirillum seropedicae* SmR1 is a nitrogen-fixing bacterium able to interact with important crops (Chubatsu et al., 2011; Pedrosa et al., 2011). The genome of this diazotroph harbors genes encoding homologs of PHA (polyhydroxyalkanoate) synthases in addition to other essential genes for PHA metabolism (Pedrosa et al., 2011; Tirapelle et al., 2013; Batista et al., 2016). The PHA synthase encoded by *phaC1* (Locus Tag: Hsero_2999; Ref seq: YP_003776395.1) is essential for poly(3-hydroxybutyrate) (PHB) synthesis in *H. seropedicae* SmR1 (Tirapelle et al., 2013; Alves et al., 2016). Other putative PHA synthase homologs present in the genome are *phaC2* (Locus Tag: Hsero_2405; Ref Seq: YP_003775812.1) and *phaC3* (Locus Tag: Hsero_0265; Ref Seq: YP_003773699.1). Phylogenetic analysis of *H. seropedicae* PhaC-like proteins (Batista et al., 2016) revealed that PhaC2 and PhaC3 group in the same clades as PhaC2 and PhaC5 from *Bradyrhizobium japonicum* USD110, respectively, both of which have no apparent role in PHB synthesis (Quelas et al., 2013). Thus *H. seropedicae* PhaC2 and PhaC3 may not be physiologically important for PHB production (Batista et al., 2016). The most common PHA produced by bacteria, in general, is PHB (Lopez et al., 2015), which is accumulated as granules in the cytoplasm under conditions of carbon excess and limitation of other elements, such as oxygen or nitrogen (Madison and Huisman, 1999). The biosynthesis of PHB relies on the central carbon metabolite acetyl-CoA. In the first step of the synthesis, two molecules of acetyl-CoA are condensed by a β-ketothiolase (PhaA), to form acetoacetyl-CoA, which is then reduced to (R)-3-hydroxybutyryl-CoA by an acetoacetyl-CoA reductase (PhaB). Finally, the (R)-3-hydroxybutyryl-CoA is polymerized by the PHA synthase (PhaC) (Rehm, 2003). Recent studies have revealed that the metabolic role of PHAs is more complex than just providing an intracellular carbon store that can be mobilized to give enhanced competitiveness to PHA-producing bacteria. To date, PHA synthesis has been correlated with protective mechanisms against a diverse range of stress conditions, such as growth in the presence of oxidants, osmotic shock, resistance to desiccation, heat, and UV irradiation (Kadouri et al., 2003; Lopez et al., 2015; Koskimäki et al., 2016; Sacomboio et al., 2017). Additionally, PHA has been suggested to be important for maintaining the redox balance of the cell (Stockdale et al., 1968; Senior et al., 1972). In this study, we have investigated the physiological consequences of PHB synthesis in *H. seropedicae* by characterizing a mutant strain lacking *phaC1*, which is unable to produce PHB (Tirapelle et al., 2013). Under conditions of oxygen limitation, we observe that this mutation influences NAD(P)H/NAD(P)^+^ ratios and increases susceptibility to oxidative stress, particularly to the superoxide propagator methyl viologen. This, in turn, impairs the activity of the redox-responsive transcriptional regulators Fnr1 and Fnr3, which are required for expression of the cytochrome c branch of the electron transport chain (ETC; Batista et al., 2013). As a consequence, the bacterium is unable to trigger effective adaptation to the shortage of oxygen and accommodate the change in redox balance. We also demonstrate that the Fnr proteins regulate the production of PHB. Thus, we have uncovered a cyclic dependency between PHB synthesis and Fnr activity that ensures reciprocal control of the redox balance under oxygen-limiting conditions.

## Materials and Methods

### Strains and Plasmids

The bacterial strains and plasmids used in this study are listed in Supplementary Table 1.

### Culture Media, Growth Conditions, and Growth Rate Determinations

The *H. seropedicae* strains were grown under 30°C in NFbHPN-Malate medium supplemented with 20 mM NH_4_Cl (Klassen et al., 1997) in two different agitation rates to ensure low aeration (120 rpm) or high aeration (350 rpm). The sensitivity of *H. seropedicae* strains to methyl viologen (a superoxide propagator) was tested using cells grown to an OD_600nm_ of 0.5, when different concentrations of methyl viologen were added to the culture. Samples were taken at regular intervals to evaluate cell growth. Control cultures without addition of a reactive oxygen species (ROS) source were run in parallel. Antibiotic concentrations used for *H. seropedicae* were 80 μg/mL for streptomycin, 500 μg/mL for kanamycin and 10 μg/mL for tetracycline. The growth rate (μ) for each condition was calculated as μ = [ln(OD_600nm_ at *t*_1_) - ln(OD_600nm_ at *t*_0_)]/(*t*_1_ -*t*_0_) using the values of turbidity measurements during the exponential phase (Chavarria et al., 2013).

### Determination of Intracellular NAD(P)H and NAD(P)^+^

Intracellular levels of reduced and oxidized NAD^+^ and NADP^+^ were determined by the improved cyclic assay (Gibon and Larher, 1997) using either ADH (Sigma #A3263) or G6PDH (Sigma #G6378), respectively. Samples were prepared from 1 mL of culture rapidly collected as described by (Nikel et al., 2008; Chavarria et al., 2013), using cells cultivated either until the mid-log (OD_600nm_ of 0.4–0.5) or late-log (OD_600nm_ of 1.0–1.2) phases. Reduced and oxidized nicotinamide adenine dinucleotides were rapidly extracted by treatment with alkali or acid, respectively, followed by extract neutralization to avoid metabolic derived changes (London and Knight, 1966; Chavarria et al., 2013). The assay was performed in 200 μL in a water bath for 30 min at 37°C. Reactions were stopped by addition of 100 μL of 5 M NaCl followed by 5 min of ice incubation. The precipitated formazan was recovered by centrifugation for 5 min at 14,000 × *g* and solubilized in 500 μL of 96% ethanol. Solubilized formazan was quantified as a function of the absorbance at 550 nm in a Biotek ELX-800 microplate reader. The standard calibration curve was set in triplicate following instructions given by Gibon and Larher (1997) using up to 30 pmol/assay of either NAD(P)H or NAD(P)^+^.

### Determination of Intracellular Adenine Nucleotides

Intracellular levels of adenine nucleotides were quantified using *H. seropedicae* strains grown as above until the mid-log phase (OD_600nm_ of 0.4–0.5). Samples were prepared using cell pellets harvested from 1.5 mL by fast centrifugation at room temperature (15 s at 12,000 × *g*). The supernatant was discarded and the microcentrifuge tubes transferred to an ice-NaCl cooling bath at -20°C. Immediately, the metabolites were extracted resuspending the pellets in 200 μL of 40:40:20 acetonitrile:methanol:water solvent system (at -20°C) with formic acid to a final concentration of 0.1 M as previously described (Bennett et al., 2008). The mixture was kept for 15 min at -20°C in an ice-NaCl cooling bath and then centrifuged for 5 min at 14,000 × *g* at 4°C for 5 min to pellet the insoluble material. The samples were analyzed by LC-MS. The chromatographic separation of metabolites was carried out injecting 10 μL of the mix in a Kinetex C18 column (Phenomenex, United States) applying a gradient with the following solvents: (A) 97:3 water:methanol with 10 mM tributylamine and 15 mM acetic acid; (B) methanol using a flow rate of 100 μL/min (Lu et al., 2010). The *m/z* values of the ions were acquired in negative mode in a quadrupole-time of flight mass spectrometer (micrOTOF-Q II, Bruker Daltonics, United States). The parameters of the ESI source were set as following: capillary voltage 4,500 V, end plate offset voltage -500 V, nebulizer pressure 2.0 bar, dry gas flow 6 L/min, drying gas temperature 180°C. The collision energy in the quadrupole was set 10 eV. The retention time for each adenine nucleotide was determined injecting standard solutions in different concentrations and monitoring the following ions: ATP, 505.9957 ± 0.05 *m/z*; ADP 426.0294 ± 0.05 *m/z*; and AMP 346.0630 ± 0.05 *m/z*.

### NMR Spectroscopy Determination of Malate and Acetate Levels in the Supernatant of Bacterial Cultures

Samples for NMR spectroscopy analysis were prepared as follows. A 200 μL of D_2_O were added to 400 μL of supernatant media from bacterial cultures after centrifugation (12,000 × *g* for 2 min). ^1^H-NMR spectra were acquired for each sample at 600 MHz (Ascend^TM^ 600, Bruker) spectrometer equipped with a 5 mm QXI inverse probe and a Sample Case autosampler. The temperature was controlled at 30°C throughout the experiment. Standard ^1^H-NMR pulse sequence with water pre-saturation (Bruker pulse program zgpr) was applied to each sample. A total of 64 transient free induction decays (FID) were collected for each experiment with a spectral width of 20 ppm. The relaxation delay was set to 1 s. The 90° pulse length was automatically calibrated for each sample at around 11 μs. The total acquisition time of each sample was 1 min 27 s. The metabolite concentrations were measured based on the height of the peaks in phosphate buffer pH 6.8: malate at δ 2.664 (CH_2_ doublet, JH → H = 14.9 Hz) and acetate at δ 1.904 (CH_3_ singlet).

### PHB Quantification

Quantification of PHB was performed by gas chromatography coupled to a flame-ionization detector essentially as described previously (Braunegg et al., 1978). In brief, acid methanolysis was performed with up to 10 mg of lyophilized bacterial cell pellets and then the derived 3-hydroxybutyric methyl ester (Me-3-HB) was dried with Na_2_SO_4_, and quantified by gas chromatography. The PHB amount in each sample was normalized by cell dry weight (cdw) and was expressed as % PHB/cdw. Alternatively, the PHB levels were quantified by Nile Red staining as described by (Zuriani et al., 2013). In brief, cells cultivated to late log phase were collected by centrifugation, resuspended in PBS (Sigma # P4417) and stained for 40 min at 30°C with 10 μg/mL of Nile Red (Sigma # 72485) prepared in DMSO. After incubation, 200 μL of cells were transferred to a 96-well black plate with clear bottom (Greiner Bio-One # 655096) and the fluorescence (λex: 485 nm and λem: 535 nm) was measured on a Tecan Infinite 200 microplate reader.

### Heme Staining

Protein extracts from bacterial cultures grown under low aeration (120 rpm) were prepared exactly as described by Batista et al. (2013). Fifty micrograms of protein were loaded without boiling onto a 15% Tris-Tricine SDS-PAGE gel, and stained using *o*-dianisidine for detection of covalently bound heme proteins (Francis and Becker, 1984).

### Analysis of Intracellular ROS Levels Using Flow Cytometry

*Herbaspirillum seropedicae* strains were grown as described above. Cells from 1 mL of culture were collected by centrifugation at 14,000 × *g* for 1 min and then resuspended in 500 μL of PBS buffer supplemented with 1 mM EDTA, 0.01% Tween 20, and 0.1% Triton X-100. Cells were subsequently incubated with 50 μM 2′,7′-dichlorodihydrofluorescein diacetate (H_2_-DCFDA) for 30 min at 30°C in the dark. Control experiments without H_2_-DCFDA addition were also set up under the same conditions. Treatments with extra ROS source or with an antioxidant agent were also performed by pre-incubation of cells with 5 mM H_2_O_2_ or with 5 mM *N*-acetyl-L-cysteine (Nac), respectively, for 30 min before addition of H_2_-DCFDA. The samples were analyzed by flow cytometry using a BD Accuri^TM^ C5 flow cytometer equipped with a 488 nm argon laser and a 533/30 nm bandpass filter (FL1-H). The median fluorescence intensity was used to determine the intracellular ROS levels.

### Analysis of Superoxide Levels by Fluorescence Microscopy

Aiming to access the levels of superoxide produced by *H. seropedicae* SmR1 and Δ*phaC1* strains, fluorescence microscopy imaging was performed after treatment of the cells with superoxide specific probe dihydroethidium (DHE). Upon entry into the cells, DHE reacts with superoxide to form oxyethidium (Zhao et al., 2003) which then interacts with nucleic acids emitting a bright red color detectable qualitatively by fluorescent microscope (Tarpey et al., 2004). Briefly, the bacterial cultures (1 mL) were harvested by centrifugation for 60 s at 10,000 × *g*, and the cell pellets were resuspended in 300 μL of PBS buffer followed by addition of 3 μL of 5 mM DHE dissolved in DMSO. After incubation for 30 min, the cells were visualized under a fluorescent microscope. The images were obtained using an Axio Imager Z2 microscope (Carl Zeiss Microscopy GmbH, Jena, Germany), equipped with four Metafer automated capture software (Metasystems GmbH, Altlussheim, Germany) and a CoolCube 1 camera (with 100× magnification). For fluorescence quantification, four images obtained from each strain (two for each biological replicate) were analyzed using the ImageJ software (Schneider et al., 2012) following the manufacturer’s instructions. Briefly, after exporting the images into ImageJ software, the images were submitted to thresholding prior to particle (cell) analysis and fluorescence intensity counting. The fluorescence intensity for each particle was normalized by its area and then the sum of the normalized fluorescence intensity of all particles (cells) was finally divided by the total number of particles in each image. The software assigned between 200 and 300 particles in each analyzed image. The results are given as the mean ± standard deviation from quantifications performed for four different images from each strain. The normalized fluorescence intensity is given in arbitrary units (AU).

### RNA Isolation and RNA-Seq Library Construction

For total RNA extraction, *H. seropedicae* SmR1 (wild-type) and Δ*phaC1* strains were grown to an optical density of approximately 1.0 (late-log phase), when high levels of PHB production occur (Tirapelle et al., 2013). Cells were collected by centrifugation and the total RNA was purified using the RNA RiboPure^TM^-Bacteria kit (Ambion^TM^) following the manufacturer’s instructions. mRNA was enriched using the MICROBExpress^TM^ kit (Ambion^TM^) performing two rounds of rRNA depletion. Up to 500 ng of mRNA enriched RNA was used for cDNA synthesis and library construction using the Ion Total RNA-Seq Kit v2 (Applied Biosystems^TM^) following the manufacturer instructions. Sample multiplexing was performed using the Ion Xpress^TM^RNA-Seq Barcode 1-16 Kit (Applied Biosystems^TM^). The RNA-Seq libraries were sequenced on the Ion Proton^TM^ (Applied Biosystems^TM^).

### Read Mapping, Differential Expression Analysis, and Other Computational Analysis

Reads were mapped against *H. seropedicae* SmR1 CDS (NC_014323) using the CLC Genomics Workbench 7.0 (CLC bio). To increase the data robustness, two statistical methods for differential expression (DE) were used. The data set corresponding to the genes differentially expressed in both methods was used for further analysis. First, the DE analysis was performed using the RPKM normalization method (Mortazavi et al., 2008) and Baggerley beta-binomial statistics included in CLC Genomics Workbench 7.0. The output table was used for data filtering considering only genes with six times coverage in at least one of the strains. DE genes were those with a fold change of at least 2, and FDR lower than 0.05. For the second DE analysis, the read counts table was exported from CLC into the RobiNA software (Lohse et al., 2012) and statistical evaluation of differential gene expression was performed by DESeq (Anders and Huber, 2010) considering a *p*-value cut-off of 0.05 using the Benjamini–Hochberg method for multiple testing corrections. Similarly, to the first analysis, genes with fold changes lower than two were excluded. The final set of regulated genes is presented in Supplementary Table 2. To check which DE genes overlapped with the *H. seropedicae* Fnr regulon, we compared the current transcriptomic dataset with the published *H. seropedicae fnr* mutant dataset (Batista et al., 2013). Additionally, potential Fnr-binding sites in the *H. seropedicae* genome were inspected by using the *E. coli* Fnr protein binding motif (Myers et al., 2013) and the FIMO search tool with default parameters (Grant et al., 2011). Finally, the list of overlapping regulated genes between both datasets was compared to those genes containing promoters with potential Fnr-binding sites. Results showing the potential direct or indirect Fnr targets are presented in Supplementary Table 3. The sequence data for all libraries analyzed in this study are available in ArrayExpress database under the accession number E-MTAB-5303.

## Results

### Accumulation of PHB Is Physiologically Important for the Synthesis of *c*-Type Cytochromes

**FIGURE 1:**
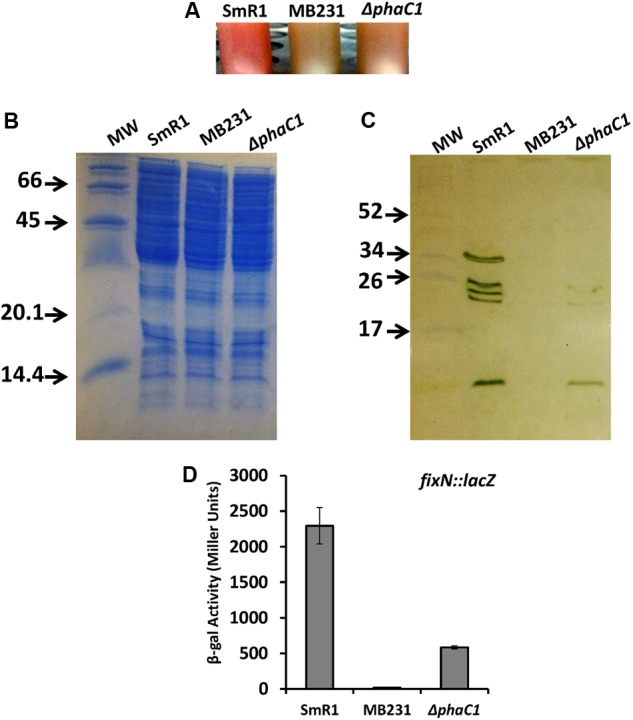
The lack of PHB production in *H. seropedicae* Δ*phaC1* influences the levels of *c*-type cytochromes. **(A)** Cell suspensions of the wild-type (SmR1), triple *fnr* mutant (MB231) and *phaC1* mutant (Δ*phaC1*) strains showing the differences in color associated with differential expression of *c*-type cytochromes. **(B)** Loading control (stained with Coomassie Brilliant Blue) from the protein extracts used for the ortho-dianisidine staining. **(C)** Levels of *c*-type cytochromes in the wild-type (SmR1), triple *fnr* mutant (MB231), and *phaC1* mutant (Δ*phaC1*) strains were analyzed after separation of protein extracts in Tris-Tricine PAGE followed by *o*-dianisidine staining for detection of covalently bound heme. The gels in panels **(B,C)** are representative of four biological replicates. The numbers in panels **(B,C)** indicate the molecular weight of protein markers in kDa. **(D)** The expression profile of the *fixN-lacZ* transcriptional reporter fusion was tested in SmR1, MB231, and Δ*phaC1* strains by measuring β-galactosidase activity according to (Miller, 1972; Batista et al., 2013). Results are the mean ± SD from three biological replicates.

In *H. seropedicae* SmR1, the production of PHB is mainly dependent upon the PHA synthase encoded by *phaC1* (formerly *phbC1*; Tirapelle et al., 2013). Quantitative and qualitative analysis of PHB production by gas chromatography (Tirapelle et al., 2013) together with fluorescence and transmission microscopy (Alves et al., 2016) revealed that a strain with an in-frame deletion of *phaC1* is unable to produce PHB. This phenotype can be complemented *in trans* by the expression of *phaC1* from either a constitutive promoter or the native promoter (Supplementary Figure 1). Furthermore, whilst the *phaC1* gene is likely to form an operon with *phaB1* (Hsero_2998), analysis of transcripts mapping to the *phaC1* (Hsero_2999) neighborhood, suggests that in-frame deletion of *phaC1* does not affect the expression of immediate downstream genes, including *phaB1* (Hsero_2998) and *phaR* (Hsero_2997) (Supplementary Figure 2; see also Supplementary Table 2). Apart from being defective in PHB production, we observed that the *phaC1* deletion strain (Δ*phaC1*) had a distinct phenotype conferred by lack of pink pigmentation when cultured in liquid media (**Figure 1A**). In *H. seropedicae* SmR1 this pigmentation is associated with *c*-type cytochromes, whose synthesis is dependent upon the redox-sensing proteins Fnr1 and Fnr3 (Batista et al., 2013). Therefore, we anticipated that the *phaC1* mutant strain would be defective in the expression of *c*-type cytochromes. To examine this hypothesis, we compared the heme-stained protein profile of the wild-type strain SmR1, with that of a triple *fnr* deletion strain (MB231) and the Δ*phaC1* strain (**Figures 1B,C**). Similar to the triple *fnr* deletion, the *phaC1* deletion strain had reduced levels of all five cytochromes when compared to the wild-type strain (SmR1). The first and third bands (from the top to the bottom of the gel) were undetectable in the protein extracts of Δ*phaC1*, while the other bands were significantly reduced (**Figure 1C**). As the synthesis of *c*-type cytochromes is mainly dependent upon the activity of the Fnr1 and Fnr3 regulatory proteins (Batista et al., 2013), it is possible that the absence of PHB imposes a physiological state that reduces transcriptional activation by Fnr proteins under oxygen-limiting conditions. To further validate the relationship between the absence of PHB and the activity of the Fnr proteins, we tested the expression of the *fixNOP* operon (encoding the *cbb_3_*-type oxidase) using a *fixN*-*lacZ* transcriptional fusion (**Figure 1D**; Batista et al., 2013). Comparing the expression profile of *fixNOP* in the strains SmR1, MB231, and Δ*phaC1* (**Figure 1D**), transcriptional activation by Fnr1 and Fnr3 was clearly affected in the Δ*phaC1* strain since the activity of the *fixN*-*lacZ* reporter was approximately 75% lower in this strain compared to the wild-type (**Figure 1D**). The reduction in *fixNOP* expression is in accordance with the absence of band 1 (FixP; Batista et al., 2013) in the heme-stained gel (**Figure 1C**). Furthermore, the reduced expression of all five *c*-type cytochromes in the Δ*phaC1* mutant also correlates with the growth penalty observed in this strain when the cells were cultured in 96-well microtiter plates, which limits oxygen diffusion and thus imposes oxygen-limiting conditions (Supplementary Figure 3).

### Deletion of the *fnr* Genes Affects PHB Production in *H. seropedicae* Under Oxygen-Limiting Conditions

**FIGURE 2:**
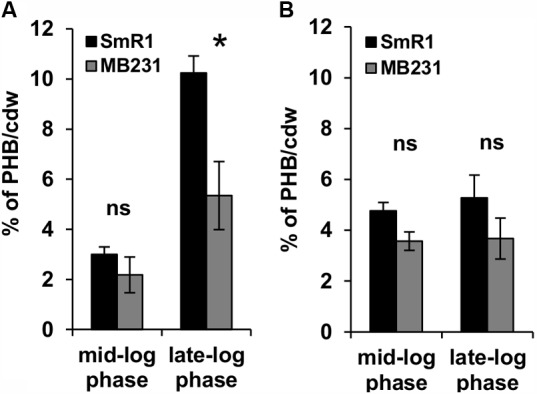
The Fnr proteins influence PHB production. PHB levels were quantified from cells grown in NFbHP-Malate medium supplemented with 20 mM NH_4_Cl, after acid methanolysis and gas chromatography of the wild-type strain (SmR1) and *phaC1* mutant (Δ*phaC1*) when cultivated under low aeration (120 rpm) **(A)** or under high aeration (350 rpm) **(B)**. Samples were taken either in the mid-log phase or the late-log phase of growth as indicated. The results are representative of two independent experiments performed in biological triplicates. ^∗^*p* < 0.01, and ns—not statistically significant (*p* > 0.05) according to an unpaired *t*-test.

Previous RNA-Seq based transcriptomic data revealed that the Fnr proteins are implicated in the expression of all three homologous PHA synthases in *H. seropedicae* (Batista et al., 2013). Transcripts mapping to the *phaC2* and *phaC3* genes were reduced by 100- and 60-fold respectively, while expression of the *phaC1* gene was reduced by approximately threefold in the triple *fnr* mutant strain compared to the wild-type (Batista et al., 2013). In addition, the biosynthesis of PHB was earlier found to be influenced by CydR, an Fnr ortholog, in the diazotroph *Azotobacter vinelandii* (Wu et al., 2001). To address whether or not the *H. seropedicae* Fnr proteins are required for PHB production, we measured PHB levels in the triple *fnr* mutant under two different phases of growth and aeration regimes (**Figure 2**). When cells were grown under low aeration (120 rpm) and harvested in the mid-log phase, no difference in PHB production was observed between SmR1 and the triple *fnr* deletion strains. However, in late-log phase when the oxygen tension in the culture was reduced, the triple *fnr* deletion exhibited a significant reduction in PHB production (approximately 50%) compared to SmR1 (**Figure 2A**). In contrast, under high aeration (350 rpm), where oxygen levels are detrimental to Fnr activity (Supplementary Figure 4), no difference in the levels of PHB was observed (**Figure 2B**), regardless of the growth phase analyzed. These observations reinforce the importance of Fnr proteins for optimal production of PHB in *H. seropedicae*.

### Increased Oxidative Stress Is Observed in the Absence of PHB Accumulation

**FIGURE 3:**
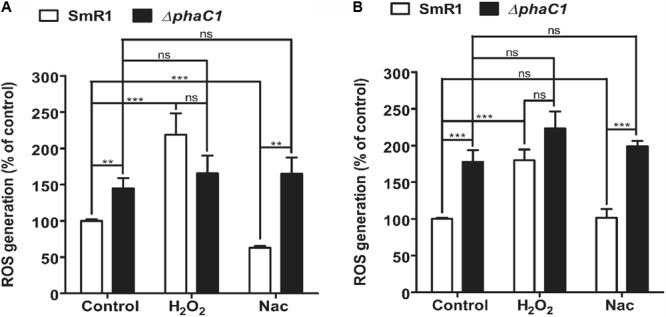
Oxidative stress is higher in the *phaC1* mutant. ROS levels were analyzed using the H_2_-DCFDA probe and flow cytometry in the wild-type strain (SmR1) and *phaC1* mutant (Δ*phaC1*) in either the mid-log phase **(A)** or the late-log phase **(B)** of growth. The control assay corresponds to measurement of ROS levels in the absence of added compounds. H_2_O_2_ and *N*-acetyl-L-cysteine (Nac) indicate respectively, treatment of cells with 5 mM hydrogen peroxide or 5 mM Nac, prior to determination of ROS levels. Data represent the average (±SEM) of two biological replicates analyzed in four technical replicates. ^∗∗^*p* < 0.001, ^∗∗∗^*p* < 0.0001, and ns—not statistically significant (*p* > 0.05) according to an unpaired *t*-test.

The decreased level of *c*-type cytochromes and the lower expression of the *fixN*-*lacZ* fusion (**Figure 1**) is apparently associated with a growth rate penalty in the *phaC1* mutant (Supplementary Figure 3), which implies that PHB accumulation is important for maintenance of Fnr activity in *H. seropedicae*. As the activity of Fnr is labile to oxygen and other ROS such as superoxide (Sutton et al., 2004; Crack et al., 2007), we were interested to know whether the *phaC1* mutant is subject to increased oxidative stress under low aeration. Using the fluorescent probe 2′,7′-dichlorodihydrofluorescein diacetate (H_2_-DCFDA), we determined that the level of intracellular ROS was approximately 50% higher in the Δ*phaC1* mutant, when cells were analyzed during the mid-log phase (OD_600_ 0.4–0.5) and was 75% higher in the late-log phase (OD_600_ 1.0–1.2) when compared with the wild-type strain (**Figures 3A,B**). After challenging the cells with 5 mM hydrogen peroxide, ROS levels increased in the wild-type strain, but did not change in the *phaC1* mutant regardless of the growth phase. We also tested the ability of both strains to mitigate the level of ROS after treatment with the chemical antioxidant Nac. This treatment enabled the SmR1 strain to reduce ROS by approximately 60% in the early-log phase (**Figure 3A**). However, in the late-log phase the antioxidant treatment did not result in relief of oxidative stress (**Figure 3B**). In contrast, for the Δ*phaC1* strain, treatment with the antioxidant was unable to alleviate ROS in either growth phases (**Figures 3A,B**). Taken together, these observations suggest that in the absence of PHB, higher levels of ROS are induced in the *phaC1* mutant strain, which then influences the activity of the Fnr proteins. Moreover, the inability of the Δ*phaC1* strain to relieve oxidative stress upon addition of a chemical antioxidant, suggests that ROS levels and the antioxidant defense mechanisms of the bacterium were saturated in both phases of growth. The increase in ROS levels in the *phaC1* mutant was observed only under oxygen limitation. When the cells were grown at 350 rpm (high O_2_), no difference in ROS was observed between the wild-type and Δ*phaC1* strains. However, upon a shift from 350 to 120 rpm (high to low O_2_) the levels of ROS in the Δ*phaC1* strain were higher than in the wild-type (Supplementary Figure 5) as observed in the assays shown in **Figures 3A,B**. In accordance with the observation that the *phaC1* deletion is subject to increased oxidative stress, we observed that *sodB* (Hsero_1165), encoding superoxide dismutase, in addition to *btuE* (Hsero_3805) and *trxB1* (Hsero_3504), encoding glutathione peroxidase and thioredoxin reductase respectively, were activated by approximately threefold in the *phaC1* mutant (Supplementary Figure 6 and Supplementary Table 2).

### Growth Inhibition by Superoxide Propagation Is More Severe in the *ΔphaC1* Strain

**FIGURE 4:**
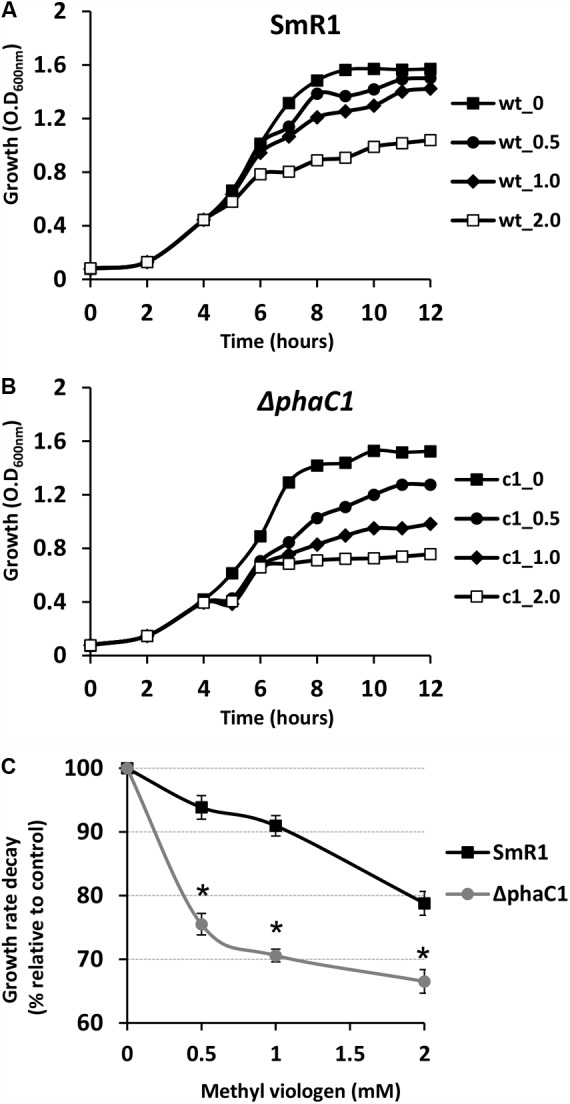
The *phaC1* mutant is more sensitive to methyl viologen. To determine the sensitivity of *H. seropedicae* strains to the superoxide generator methyl viologen, the wild-type **(A)** and the *phaC1* mutant **(B)** strains were cultured in liquid media to an OD_600nm_ of 0.5, when different concentrations of superoxide were added. In panels **(A,B)**, the closed squares indicate the growth of the strains without methyl viologen addition, while the circles, diamonds and open squares, indicate the growth profile upon addition of 0.5, 1.0 and 2.0 mM of methyl viologen, respectively. In panel **(C)**, the percentage of growth rate decay between the wild-type (black lines—squares) and *phaC1* mutant (gray lines—circles) is shown. Typical growth rates (100%) were 0.46 ± 0.003 /h for the wild-type and 0.43 ± 0.002 /h for the *phaC1* mutant in the absence of methyl viologen. The data is representative of three independent biological replicates. ^∗^*p* < 0.0001 according to an unpaired *t*-test.

**FIGURE 5:**
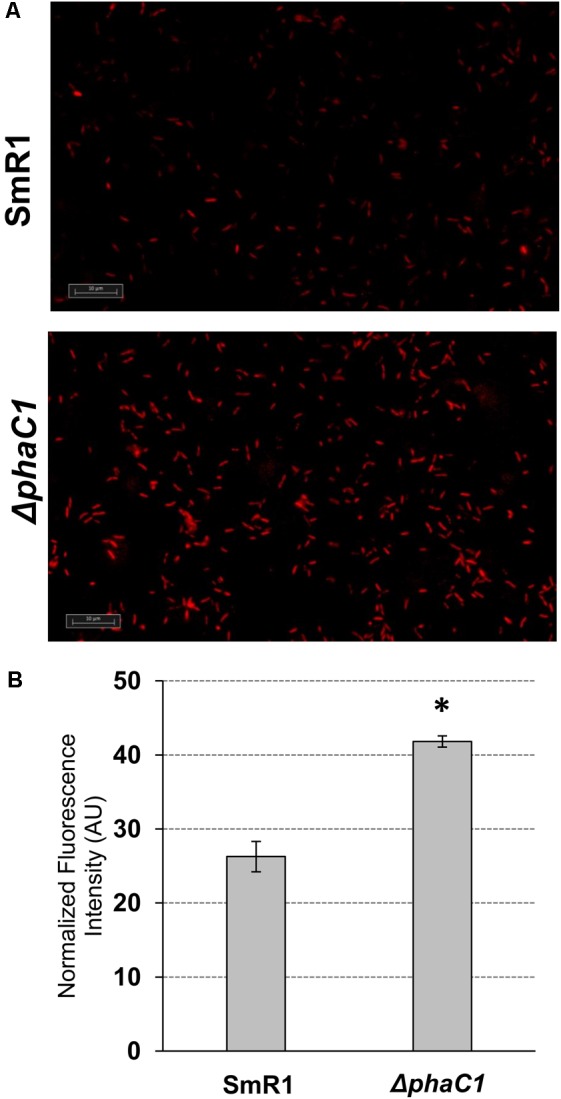
The *H. seropedicae* Δ*phaC1* strain produces more superoxide than the wild-type (SmR1). Cells were grown in NFbHP-Malate media supplemented with 20 mM of NH_4_Cl until the late-log phase (OD_600nm_ = 1.0), treated with the superoxide specific probe DHE and then visualized by fluorescence microscopy as described in Section “Materials and Methods.” In panel **(A)**, one representative image for each strain is shown. In panel **(B)**, the relative fluorescence quantification is shown. The quantitative analysis was made as described in Section “Materials and Methods.” The results are given as the mean ± standard deviation from quantifications performed for four different images from each strain (two for each biological replicate). AU indicates arbitrary units of normalized fluorescence intensity. ^∗^*p* < 0.0001 according to a two-sample *t*-test.

**FIGURE 6:**
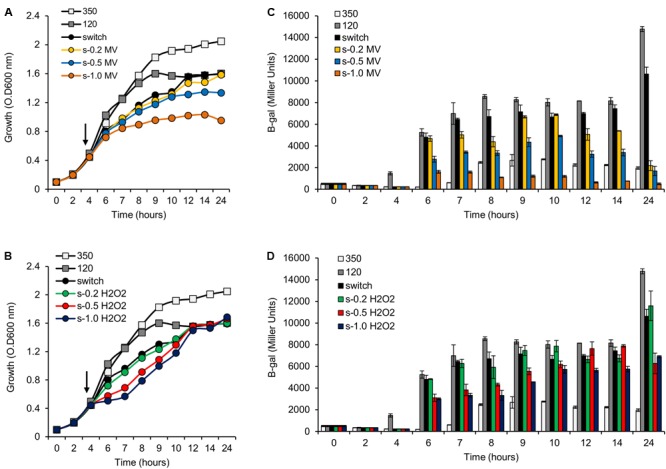
Activity of the *pfixN-lacZ* reporter fusion upon addition of methyl viologen (MV) or H_2_O_2_. **(A)** Representative growth profile of *H. seropedicae* SmR1 upon addition of MV. Samples were collected at the indicated times for measuring the *pfixN-lacZ* expression in panel **(C)**. **(B)** Representative growth profile of *H. seropedicae* SmR1 upon addition of H_2_O_2_. Samples were collected at the indicated times for measuring the *pfixN-lacz* activity in panel **(D)**. **(C,D)** Activity of the Fnr-dependent transcriptional fusion *pfixN-lacZ* was assayed upon addition of MV or H_2_O_2_ respectively. The arrows, in panels **(A,B)**, indicate the addition of different amounts of MV or H_2_O_2_ as indicated. The color codes for symbols and bars in the graphs are as follows: white, growth under high aeration or 350 rpm (350); gray, growth under low aeration or 120 rpm (120); black, growth upon switch from 350 to 120 rpm (switch); yellow, switch from 350 to 120 rpm and addition of 0.2 mM of MV (s-0.2 MV); blue, switch from 350 to 120 rpm and addition of 0.5 mM of MV (s-0.5 MV); orange, switch from 350 to 120 rpm and addition of 1.0 mM of MV (s-1.0 MV); green, switch from 350 to 120 rpm and addition of 0.2 mM of H_2_O_2_ (s-0.2 H_2_O_2_); red, switch from 350 to 120 rpm and addition of 0.5 mM of H_2_O_2_ (s-0.5 H_2_O_2_); dark blue, switch from 350 to 120 rpm and addition of 1.0 mM of H_2_O_2_ (s-1.0 H_2_O_2_).

The results above demonstrate that mutation of the *phaC1* gene increases oxidative stress when cells are grown in laboratory conditions. However, in the natural environment, this bacterium would have to cope with external sources of ROS generated either by plants or by other competing organisms in the rhizosphere (Nanda et al., 2010; Sharma et al., 2012; Koskimäki et al., 2016). We therefore evaluated the growth phenotype of both wild-type and Δ*phaC1* strains when an extra source of ROS was added to the culture media. When different concentrations of methyl viologen, a generator of intracellular superoxide (Gaudu and Fontecave, 1994), was added to the cultures, the *phaC1* mutant had a reduced growth rate compared to the wild-type (**Figure 4**). However, when a similar treatment was performed using H_2_O_2_ as an alternative ROS source, no differences in growth rates between both strains were observed (Supplementary Figure 7). These observations imply that oxidative damage generated in the absence of PHB is perhaps related to increased levels of intracellular superoxide production. To gain further physiological support for this hypothesis, we compared the levels of superoxide production in the wild-type and Δ*phaC1* strains by staining the cells with the superoxide specific probe DHE, followed by fluorescence microscopy visualization, as described in Section “Materials and Methods.” The fluorescence micrographs of the stained cells, clearly suggest that the levels of superoxide are higher in the Δ*phaC1* strain (**Figure 5**). In addition, cell suspensions of the Δ*phaC1* strain showed brighter red fluorescence under UV-light when stained with DHE (Supplementary Figure 8), further supporting our hypothesis. Finally, considering that superoxide amplifies the oxidation of [4Fe-4S]^2+^ clusters (Sutton et al., 2004; Crack et al., 2007), we were interested to know whether additional levels of superoxide can affect the activity of the Fnr proteins in the wild-type strain. To examine this, we assayed the activity of the *fixN*-*lacZ* transcriptional fusion, which is dependent upon both Fnr1 and Fnr3, following the addition of either methyl viologen or H_2_O_2_ to the wild-type strain (**Figure 6**). While both oxidants were observed to reduce Fnr activity, the effect of methyl viologen was more pronounced, as the addition of 1.0 mM of methyl viologen was sufficient to reduce Fnr activity to similar levels to those observed in the Δ*phaC1* mutant (**Figures 1, 6**). Altogether, these observations imply that increased levels of superoxide in the Δ*phaC1* mutant correlate with inactivation of the Fnr proteins, as suggested by the results shown in **Figure 1**.

### The Redox Balance and the Energy Status Are Perturbed in the *phaC1* Deletion Strain

**FIGURE 7:**
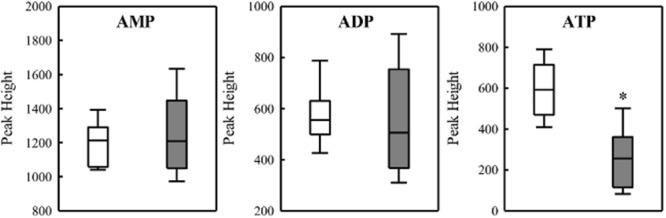
Levels of adenine nucleotides in the wild-type SmR1 and Δ*phaC1* strains. The metabolites were analyzed by LC-MS as described in Section “Materials and Methods” and the peak heights were compared. Box plots represent the data of three biological replicates analyzed in three technical replicates. ^∗^*p* < 0.00001 according to a two-sample *t*-test.

To investigate the relationship between PHB production and redox balance, we determined the levels of the pyridine nucleotide pool in the Δ*phaC1* and wild-type strains during the mid-log and late-log phases of growth, corresponding to an OD_600nm_ of 0.4–0.5 and 1.0–1.2, respectively, using cells cultured at 120 rpm (**Table 1**). During the mid-log phase, both NADPH/NADP^+^ and NADH/NAD^+^ ratios were increased by approximately two- and threefold, respectively, in the Δ*phaC1* strain when compared to the wild-type. During the late-log phase, the NADH/NAD^+^ ratio also increased in the Δ*phaC1* strain (approximately threefold). In contrast, a slight decrease in the NADPH/NADP^+^ ratio was observed in the Δ*phaC1* strain. The data presented in **Table 1** also reveal that the wild-type strain of *H. seropedicae* is able to undergo physiological adaptations to keep the NAD(P)H/NAD(P)^+^ ratios almost constant during both the exponential and the late-log phase. In contrast, more significant fluctuations in the NAD(P)H/NAD(P)^+^ ratios were observed in the *phaC1* mutant when pyridine nucleotide levels were compared in the two growth phases. The inability of the *phaC1* mutant to maintain the NAD(P)H/NAD(P)^+^ ratios, exemplified by increased levels of reduced pyridine nucleotides (**Table 1**) may account for the exacerbated sensitivity of the mutant to oxidative stress (**Figures 3, 4**). In addition, we also investigated the levels of adenine nucleotides in both the *phaC1* mutant and wild-type strains by measuring the levels of AMP, ADP, and ATP as described in Section “Materials and Methods.” Interestingly ATP levels in the *phaC1* mutant were reduced by 2.3-fold when compared to the wild-type, while the levels of both ADP and AMP remained unaltered in both strains (**Figure 7**).

**Table 1:** Effect of the *phaC1* mutation on pyridine nucleotide pools at different growth phases.

	Mid-log phase		Late-log phase	
Nucleotide pool	Wild-type	Δ*phaC1*	WT	Δ*phaC1*
NADH	1.68 ± 0.28^a^	2.31 ± 0.26	0.64 ± 0.10	1.74 ± 0.16
NAD^+^	29.38 ± 3.57	21.07 ± 2.95	13.48 ± 1.10	11.31 ± 2.26
NADH/NAD^+^	0.06	0.11	0.05	0.15
NADPH	2.22 ± 0.28	3.63 ± 0.68	1.32 ± 0.22	1.53 ± 0.15
NADP^+^	5.58 ± 0.55	2.90 ± 0.41	2.69 ± 0.46	3.83 ± 0.31
NADPH/NADP^+^	0.40	1.25	0.49	0.40

*^*a*^pmol/μg/protein ± SD of four assays performed in biological replicates.*

### Transcriptional Profiling Indicates That Deletion of *phaC1* Influences Expression of the Fnr Regulon

To examine the effect of the *phaC1* mutation on gene expression, we carried out RNA-Seq based transcriptome analysis using late log-phase cultures of the Δ*phaC1* and wild-type strains, when the peak of PHB production is observed under our experimental conditions (Tirapelle et al., 2013). Analysis of transcripts mapping to *H. seropedicae* revealed that 678 genes were differentially expressed (Supplementary Table 2). From these, 276 were downregulated, while 402 were upregulated in the *phaC1* mutant strain. Approximately 60 of the differentially expressed genes are direct or indirect targets for Fnr1 and Fnr3 proteins (Supplementary Table 3) previously identified in the transcriptome analysis of the triple *fnr* mutant strain (Batista et al., 2013). Some of these genes are likely to be directly regulated by Fnr, since Fnr binding motifs were found upstream of the promoters using a FIMO motif search (Grant et al., 2011). The Fnr regulon in *H. seropedicae* comprises 187 genes (Batista et al., 2013), with the *phaC1* mutation influencing about 30% of these genes.

### Metabolic Changes Triggered in the *phaC1* Mutant

**FIGURE 8:**
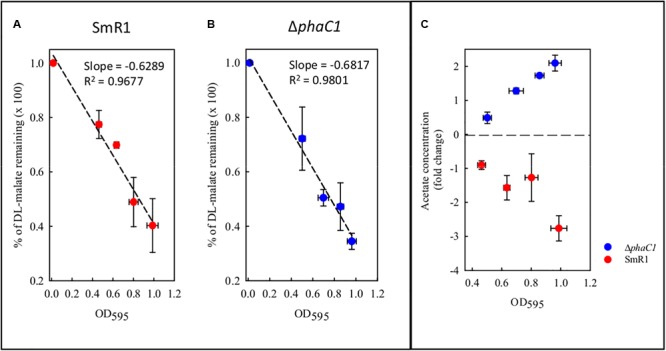
Levels of malate and acetate detected in the supernatant of cultures from the wild-type SmR1 and Δ*phaC1* strains. Both metabolites were quantified using NMR spectroscopy as described in Section “Materials and Methods.” In panels **(A,B)**, the time course of malate consumption during different stages of growth are shown in the wild-type (SmR1) and Δ*phaC1* strains, respectively. In panel **(C)**, the relative levels of acetate detected in the supernatants of the wild-type (red dots) and Δ*phaC1* (blue dots) are shown.

During the colonization of a given environment, limitations in oxygen availability may be encountered as a consequence of cell density increases and the metabolic activity of competing organisms. Under such conditions decreased electron flux through the ETC may occur, with concomitant reduction in the consumption of reducing equivalents. To avoid the stress generated by imbalanced levels of reducing equivalents, the wild-type strain diverts acetyl-CoA from the TCA cycle and NAD(P)H from the ETC into the biosynthesis of PHB. As the *phaC1* mutant is unable to operate this mechanism, we tried to address how excessive accumulation of acetyl-CoA and NAD(P)H is avoided in the absence of PHB production, by quantifying metabolites in culture supernatants. Surprisingly, we found that the consumption of malate from the culture media does not differ between the wild-type and Δ*phaC1* strains during various stages of growth (**Figure 8**). However, we observed that acetate levels in culture supernatants were approximately twofold higher in the *phaC1* mutant strain when compared to the wild-type (**Figure 8**).

## Discussion

In this study, we have determined the physiological importance of PHB for maintenance of the redox equilibrium in *H. seropedicae*. Transcription profiling of the Δ*phaC1* mutant together with further physiological characterization suggests that in the absence of PHB synthesis, oxidative stress is exacerbated by increased levels of superoxide. This, in turn, correlates with partial inactivation of the Fnr proteins. The imbalance in the NAD(P)H/NAD(P)^+^ ratios observed in the *phaC1* mutant is likely to be responsible for increased generation of superoxide, either by Complex I (Hirst et al., 2008; Pryde and Hirst, 2011) or Complex III (Lanciano et al., 2013). In *Rhodobacter sphaeroides*, the rate of superoxide generation by Complex III is modulated by the universal stress protein UspA (Su et al., 2015). Interestingly, a gene encoding a homolog of *R. sphaeroides* UspA (Hsero_2299) was downregulated 10-fold in the *phaC1* mutant (Supplementary Table 2). Additionally, the decreased level of ATP in the *phaC1* mutant concurs with our observation that this mutant has reduced levels of *c*-type cytochromes (**Figure 1**), which are important for fine-tuning the electron flux in the respiratory chain, in order to maintain normal levels of ATP synthesis and facilitate growth under oxygen-limiting conditions.

Our results show that in *H. seropedicae*, and most likely in other PHA-producing bacteria, biosynthesis of PHB is used to divert acetyl-CoA and NAD(P)H from the TCA cycle and the ETC, respectively, thus serving as an alternative electron sink to allow the elimination of excess acetyl-CoA and reducing equivalents. How exactly the partitioning mechanism between TCA cycle and PHB biosynthesis operates has yet to be elucidated, but it is likely that a combination of factors controlling acetyl-CoA/CoA and NAD(P)H/NAD(P)^+^ ratios, in addition to maintenance of the TCA cycle are major players in this mechanism (Trainer and Charles, 2006). In *H. seropedicae* SmR1, the aconitase enzyme was found to interact with PHB granules (Tirapelle et al., 2013). Perhaps, sequestration of aconitase during PHB production might control TCA cycle activity, thus helping to operate the partitioning mechanism. The *phaC1* mutant is apparently unable to operate this partitioning mechanism, resulting in increased levels of acetate excretion. Although acetate overflow may help to drain carbon, it does not apparently relieve the redox imbalance that generates oxidative stress.

Expression of protein complexes important for both the synthesis and activity of the cytochrome *c* branch of the ETC is dependent upon the Fnr1 and Fnr3 proteins in *H. seropedicae* SmR1 (Batista et al., 2013). Additionally, Fnr proteins are required for transcriptional activation of all three genes encoding PHA synthases in this organism (Batista et al., 2013). Therefore, it is feasible that regulation of the redox balance is primarily controlled by the Fnr proteins, since optimal PHB production and respiration at low oxygen concentrations are dependent upon Fnr. Conversely, the activation of PHB synthesis as an electron sink is necessary to prevent oxidative stress and consequent impairment of Fnr-dependent transcriptional regulation. Thus, PHB synthesis and maintenance of Fnr activity exhibit cyclic dependency in *H. seropedicae*, since transcriptional activation by Fnr proteins is essential for full levels of PHB synthesis, whilst production of PHB is essential to prevent inactivation of Fnr. Recently, another link between PHB synthesis and transcriptional regulation by Fnr-like proteins was suggested in *Sinorhizobium meliloti* Rm1021. It was found that different mutants deficient in PHB synthesis and accumulation exhibited reduced transcript levels of various genes associated with the presence of an Fnr-like regulatory sequence in their respective promoters (D’Alessio et al., 2017). It is therefore conceivable that a similar cyclic dependence between PHB synthesis and activity of Fnr-like proteins occurs in *S. meliloti* Rm102, to link the control of the PHB cycle with the activity of redox-sensitive regulatory circuits.

The influence of PHB synthesis on the Fnr regulon under conditions of oxygen limitation could perhaps provide a mechanism for shutting down expression of superoxide-generating components of the ETC when there is insufficient carbon available to divert electrons into PHB synthesis. The use of an electron sink appears to be an important mechanism for *H. seropedicae* to adapt to changes in the redox status imposed by the environment. For example, in the presence of nitrate as nitrogen source, the respiratory nitrate reductase (NAR—encoded by *narGHJI* operon), is activated by the Fnr1 and Fnr3 proteins (Batista et al., 2013; Bonato et al., 2016). The reduction of nitrate by NAR also appears to be used as an electron sink in addition to PHB synthesis, since *H. seropedicae* is unable to respire nitrate under anaerobiosis (Bonato et al., 2016).

The findings reported here regarding the physiological significance of PHB together with the observation that the *phaC1* mutant has decreased competitiveness during plant colonization (Balsanelli et al., 2015) indicates that PHB plays an important role in cellular metabolism, not only under free-living conditions but also during the *Herbaspirillum–*plant interaction. PHB may be required to support redox homeostasis, thus allowing efficient bacterial competition in the rhizosphere and increased fitness for the early stages of the interaction with crops. In *H. seropedicae* and possibly other PHA-producing bacteria, the link between redox equilibrium and PHB synthesis is tightly controlled. Interestingly, it was recently found that increased levels of NADPH in an *ntrC* mutant of *H. seropedicae* led to higher PHB production and increased fitness toward oxidative stress (Sacomboio et al., 2017). Finally, the transcriptional changes uncovered here, together with increased knowledge of PHB metabolism in *H. seropedicae*, may enable engineering of more efficient PHB-producing strains, with potential biotechnological applications, including production of biodegradable plastics and development of bacterial inoculants with improved competitiveness in the rhizosphere.

## Author Contributions

MB, CT, and MM-S designed and performed experiments, analyzed the data, and wrote the manuscript. MZS prepared and sequenced RNA-Seq libraries. LA performed PHB quantifications and the fluorescence microscopy. GV performed ROS quantification experiments. GS performed metabolite quantification by NMR spectroscopy. FOP, MBS, and ES conceived the idea of the project and analyzed the data. RD and MM-S supervised the study, analyzed the data, and wrote the manuscript.

## Conflict of Interest Statement

The authors declare that the research was conducted in the absence of any commercial or financial relationships that could be construed as a potential conflict of interest.
