# Supplementary material for: PHB Biosynthesis Counteracts Redox Stress in Herbaspirillum seropedicae
Source: Front Microbiol. 2018 Mar 15;9:472. doi: 10.3389/fmicb.2018.00472 (PMC5862806; doi:10.3389/fmicb.2018.00472)
Supplement: Supplementary file 3 [file Data_Sheet_1.DOCX]

Supplementary Material

**PHB biosynthesis counteracts the redox stress in *Herbaspirillum seropedicae***

**Marcelo Bueno Batista, Cícero Silvano Teixeira, Michelle Zibetti Tadra Sfeir, Luís Paulo Silveira Alves, Glaucio Valdameri, Fábio de Oliveira Pedrosa, Guilherme Lanzi Sassaki, Maria Berenice Reynaud Steffens, Emanuel Maltempi de Souza, Ray Dixon and Marcelo Müller-Santos^*^**

*** Correspondence:** Marcelo Müller-Santos; e-mail: [marcelomuller@ufpr.br](mailto:marcelomuller@ufpr.br)

# Supplementary Data

For accessing all information of the analysed transcriptomic libraries, please refer to the ArrayExpress database under the accession number E-MTAB-5303.

# Supplementary Figures and Tables

## Supplementary Figures


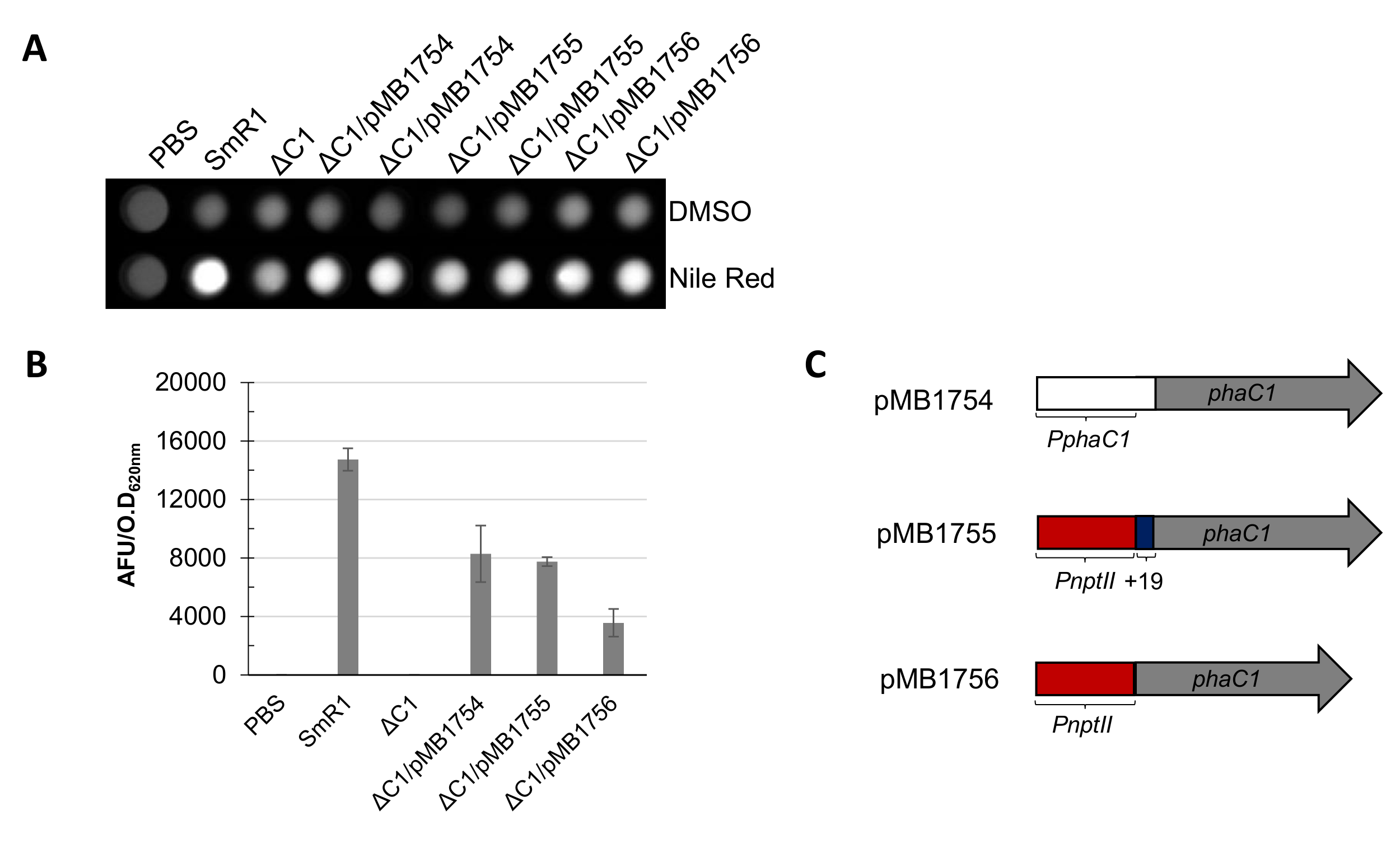


**Supplementary Figure 1** – Production of PHB in the *phaC1* mutant is restored by *in trans* complementation. The PHB levels were quantified by Nile Red staining as described by ([Zuriani et al., 2013](#_ENREF_7)). In brief, cells cultivated to late log phase were collected by centrifugation, resuspended in PBS (Sigma # P4417) and stained for 40 minutes at 30^o^C with 10 µg/mL of Nile Red (Sigma # 72485) prepared in DMSO. After incubation, 200 µL of cells were transferred to a 96 well black plate with clear bottom (Greiner Bio-One # 655096), and the fluorescence (λ_ex_: 485 nm and λ_em_: 535 nm) was measured on a Tecan Infinite200 microplate reader. In A, a representative image of the stained cells visualised under UV-light (312 nm) is shown. The upper row is the negative staining control containing cells (or PBS only) and DMSO, whereas the lower row shows the cells stained with Nile Red as above. In B, the fluorescence quantification, given as AFU (Abritary Fluorescence Units) is shown. The AFU was normalised by the cell density (OD_620 nm_). Results are representative of a biological triplicate. In C, a schematic representation of constructs used for complementation is shown. Further information about the constructs can be found in Supplementary Table 1.

**Supplementary Figure 2**


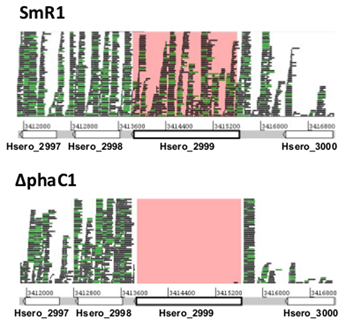


**Supplementary Figure 2.** *In frame* deletion of *phaC1* (Hsero_2999) does not affect the expression of the downstream genes *phaB1* (Hsero_2998) and *phaR* (Hsero_2997). The read mapping from transcriptional profiling of SmR1(upper panel) and Δ*phaC1* (lower panel) were visualized using Artemis ([Rutherford et al., 2000](#_ENREF_4)).

**Supplementary Figure 3**


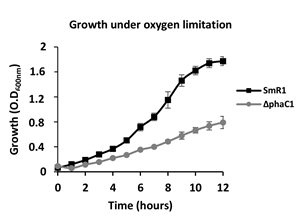


**Supplementary Figure 3**. The *H. seropedicae phaC1* mutant strain suffers a growth rate penalty under oxygen limitation when compared to the wild type (SmR1). Growth was assayed using cell suspensions prepared in NFb-MalateHP supplemented with 20 mM of NH4Cl, to an initial OD_600 nm_ of 0.05. After preparation, 200 µL of cell suspension were distributed in 96-well microplates (CELLSTAR^®^ – Greiner Bio-One #655180) and incubated at 30ºC and 120 rpm. The plates were covered with adhesive plate sealer to avoid excessive evaporation. Every hour the OD_600 nm_ was measured using a microplate reader. The microplate cultivation limits the oxygen diffusion enabling the cultures to become more oxygen limited when compared to cultivation in conical flasks.

**Supplementary Figure 4**


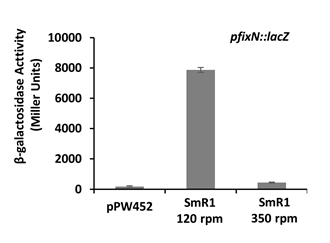


**Supplementary Figure 4**. The *H. seropedicae* Fnr proteins are not active under high aeration (350 rpm). The expression profile of the *fixN-lacZ* transcriptional reporter fusion was tested in the wild-type strain (SmR1) cultured under low (120 rpm) or high aeration (350 rpm). The promoterless vector (ppw452) was used as a control. The β-galactosidase assay was performed according to ([Miller, 1972](#_ENREF_3); [Batista et al., 2013](#_ENREF_1)). Results are the mean ± SD from three biological replicates.

**Supplementary Figure 5**


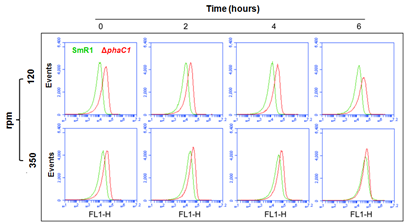


**Supplementary Figure 5**. ROS levels are higher in the Δ*phaC1* mutant strain only under low oxygen conditions. Both SmR1 (green lines) and Δ*phaC1* (red lines) strains were cultured under high aeration rate (350 rpm), to ensure highly oxic conditions, until an OD_600_ of 0.4 was reached and were then switched to low aeration (120 rpm), aiming to achieve low oxygen conditions. The time indicated is related to the period of ROS measurement that elapsed after the switch to 120 rpm (upper panel). Control cultures were analysed in parallel maintained under high aeration (350 rpm - lower panel)

**Supplementary Figure 6**


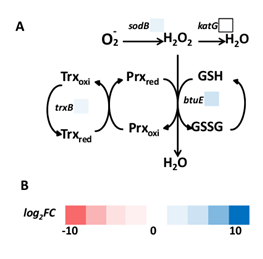


**Supplementary Figure 6**. Genes related to ROS defence mechanisms are activated in the phaC1 mutant. A scheme of the main components of the ROS defence pathway is shown (A). The colour-coded boxes along each gene in A correspond to the log_2_ fold change (log_2_FC) levels according to the scale in (B).

**Supplementary Figure 7**


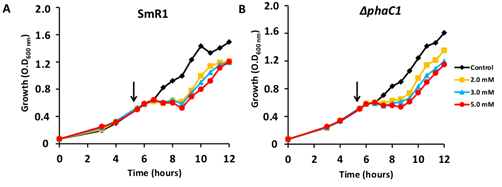


# Supplementary Figure 7. *H. seropedicae* SmR1 (wild type) and the Δ*phaC1* mutant have similar growth profiles upon H_2_O_2_. The sensitivity of SmR1 (wild type) and Δ*phaC1* strains were compared by determining the effect of the addition of H_2_O_2_ on growth in NFb-MalateHP supplemented with 20 mM of NH_4_Cl. The arrow indicates the time of H_2_O_2_ addition using the concentrations indicated in the figure legend (right panel). The control experiment (black lines and symbols) was performed without addition of H_2_O_2_. Data are representative of two independent biological replicates.

**Supplementary Figure 8**


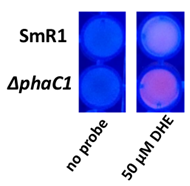


**Supplementary Figure 8**. The levels of superoxide are higher in the *ΔphaC1* strain. Cells (1 mL) were harvested by centrifugation, resuspended in 300 µL of PBS buffer and subsequently incubated with 50 µM of DHE as described in the experimental procedures. After 1-hour incubation, 200 µL of cells were spotted onto wells of a 96-well microplate (CELLSTAR^®^ – Greiner Bio-One #655180) and photographed under UV-light.

**3. Supplementary Tables**

# Supplementary Table 1. Strains and plasmids used in this study

| **Strains / plasmids** | **Relevant characteristic** | **Reference** |
| --- | --- | --- |
| SmR1 | *Herbaspirillum seropedicae* Z78 but Sm^R^ 100µg/mL, Nif^+^ | ([Souza et al., 2000](#_ENREF_5)) |
| Δ*phaC1* | *phaC1* deletion mutant derived from *H. seropedicae* SmR1 | ([Tirapelle et al., 2013](#_ENREF_6)) |
| MB231 | Triple *fnr* mutant from *H. seropedicae* SmR1 | ([Batista et al., 2013](#_ENREF_1)) |
| pPWPFN | Tc^R^, Mob, *fixNOP* promoter fused to *lacZ* gene | ([Batista et al., 2013](#_ENREF_1)) |
| pFAJ1700 | Tc^R^ and Amp^R^. Promoterless expression vector and RK2 par locus, replicative in Gram-negative bacteria | ([Dombrecht et al., 2001](#_ENREF_2)) |
| pFAJ1708 | Tc^R^ and Amp^R^. *nptII* constitutive promoter and RK2 par locus, replicative in Gram-negative bacteria | ([Dombrecht et al., 2001](#_ENREF_2)) |
| pMB1754 | Derived from pFAJ1700. RK2 par, Tc^R^ and Amp^R^. Hs-*phaC1* and its own promoter as a 2560 bp amplified fragment inserted into XbaI/EcoRI linearized pFAJ1700 by isothermal assembly. | This study |
| pMB1755 | Derived from pFAJ1708. RK2 par, Tc^R^ and Amp^R^. Hs-*phaC1+19* (N-termini extended by 19 amino acids) as an 1824 bp amplified fragment inserted into XbaI/EcoRI linearized pFAJ1708 by isothermal assembly using. Expression guided by the *nptII* promoter. | This study |
| pMB1756 | Derived from pFAJ1708. RK2-par, TcR and AmpR. *Hs-phaC1* (original annotation) as a 1767 bp amplified fragment inserted into XbaI/EcoRI linearized pFAJ1708 by isothermal assembly using. Expression guided by the *nptII* promoter. | This study |

**Supplementary Table 2** and **3** containing transcriptomic data were uploaded apart.

**References**

Batista, M.B., Sfeir, M.Z., Faoro, H., Wassem, R., Steffens, M.B., Pedrosa, F.O., Souza, E.M., Dixon, R., and Monteiro, R.A. (2013). The *Herbaspirillum seropedicae* SmR1 Fnr orthologs controls the cytochrome composition of the electron transport chain. *Sci Rep* 3**,** 2544. doi: 10.1038/srep02544.

Dombrecht, B., Vanderleyden, J., and Michiels, J. (2001). Stable RK2-derived cloning vectors for the analysis of gene expression and gene function in gram-negative bacteria. *Mol Plant Microbe Interact* 14**,** 426-430. doi: 10.1094/mpmi.2001.14.3.426.

Miller, J.H. (1972). "Experiments in Molecular Genetics." (New York: Cold Spring Harbor Laboratory Press).

Rutherford, K., Parkhill, J., Crook, J., Horsnell, T., Rice, P., Rajandream, M.-A., and Barrell, B. (2000). Artemis: sequence visualization and annotation. *Bioinformatics* 16**,** 944-945. doi: 10.1093/bioinformatics/16.10.944.

Souza, E.M., Pedrosa, F.O., Rigo, L.U., Machado, H.B., and Yates, M.G. (2000). Expression of the nifA gene of *Herbaspirillum seropedicae*: role of the NtrC and NifA binding sites and of the -24/-12 promoter element. *Microbiology* 146 ( Pt 6)**,** 1407-1418. doi: 10.1099/00221287-146-6-1407.

Tirapelle, E.F., Muller-Santos, M., Tadra-Sfeir, M.Z., Kadowaki, M.A., Steffens, M.B., Monteiro, R.A., Souza, E.M., Pedrosa, F.O., and Chubatsu, L.S. (2013). Identification of proteins associated with polyhydroxybutyrate granules from *Herbaspirillum seropedicae* SmR1 - old partners, new players. *PLoS One* 8**,** e75066. doi: 10.1371/journal.pone.0075066.

Zuriani, R., Vigneswari, S., Azizan, M.N.M., Majid, M.I.A., and Amirul, A.A. (2013). A high throughput Nile red fluorescence method for rapid quantification of intracellular bacterial polyhydroxyalkanoates. *Biotechnology and Bioprocess Engineering* 18**,** 472-478. doi: 10.1007/s12257-012-0607-z.
